# Gastro-Intestinal Symptoms in Palliative Care Patients

**DOI:** 10.3390/curroncol31040174

**Published:** 2024-04-21

**Authors:** Golda Elisa Tradounsky

**Affiliations:** 1 Palliative Care Services, Mount Sinai Hospital, CIUSSS West Central of the Island of Montreal, 5690 Cavendish Blvd, Côte Saint-Luc, QC H4W 1S7, Canada; golda.tradounsky.med@ssss.gouv.qc.ca; 2 Department of Family Medicine, McGill University, Montréal, QC H3S 1Z1, Canada

**Keywords:** palliation, constipation, nausea and vomiting, bowel obstruction, ascites, bleeds

## Abstract

This review of the palliation of various gastro-intestinal (GI) symptoms encountered in cancer patients is by no means exhaustive. Frequent symptoms such as constipation, nausea and vomiting, bowel obstructions, ascites and bleeds will be discussed, focusing on their assessment and most importantly, how to control the associated symptoms. All of these symptoms and GI complications can significantly impact patients’ quality of life (QOL) and should be treated as quickly and aggressively as possible.

## 1. Introduction

Gastro-intestinal (GI) symptoms occur frequently in palliative care patients and have a significant impact on their quality of life (QOL). Oncologists, family physicians, nurse practitioners and nurses have an important role in the care of these patients; early detection and treatment of these GI disturbances can help alleviate further discomfort and burden for the patients and their family. This article will do a cursory review of assessment and treatment of constipation, nausea and vomiting, bowel obstructions, GI bleeds and ascites. It is by no means an exhaustive review, as patients can experience other GI symptoms than the ones listed above. PubMed (https://pubmed-ncbi-nlm-nih-gov.proxy3.library.mcgill.ca/?otool=icamuhslib, accessed on 1 November 2023) was used for capturing of the last 5 years of English literature on various subjects such as constipation in palliative care or palliation, nausea and vomiting, bowel obstruction, GI bleeds and ascites. Earlier articles were retained if these seemed to contribute to pertinent substantial recommendations. To note as well is that though this article focuses on symptomatic cancer patients, family physicians and primary care nurse practitioners will also be addressing the palliative needs of patients with non-malignant terminal illnesses; some of the following recommendations may apply to that population as well.

## 2. Constipation

Constipation occurs in 23–87% of patients with an advanced illness [1,2]. It causes much discomfort; at a minimum there can be a sensation of bloating but it can lead to nausea, anorexia, pain, overflow diarrhea, urinary retention and delirium. There are many risk factors in patients with a terminal illness that can lead to constipation: immobility, decreased fluid intake, decreased intake of foods and fibre, lack of privacy, ascites, diabetic neuropathy, hypercalcemia and various medications namely some anti-depressants, opioids, diuretics, calcium-channel blockers, antipsychotics such as olanzapine and 5HT3 serotonin receptor blocker anti-emetics (ondansetron).

One assesses for constipation by obtaining a thorough history of passage of stool. A frequency of three or more bowel movements (BM) per week falls into the norm. The consistency should be firm to soft. The size of the stool should be greater than small. Straining, feeling of incomplete evacuation and pain on defecation (from fissures or hemorrhoids) should all be seen as possible signs of constipation. Tools such as the Victoria Hospice Bowel Performance Scale or Bristol Stool Form Scale can help patients and healthcare providers track the laxation problem. A physical examination of the abdomen looking for bowel sounds, distension and masses (stool should be depressible), might help to distinguish constipation from a bowel obstruction. If the patient is not immunocompromised, a rectal exam (DRE) will elucidate whether there are fissures or hemorrhoids which can worsen the constipation issue, as well as detect stool in the rectum or an obstructing mass. Also of note is that an empty dilated rectum could indicate the presence of a fecaloma in the segment above. A simple investigation is a radiograph of the abdomen. The author uses this in case of diarrhea to ascertain that it is overflow diarrhea. The X-ray can be assessed by dividing the abdomen into four quadrants: the ascending, transverse, descending colons and rectosigmoid quadrants. Each quadrant can be assigned a number from 0 to 3, where 0 means there is no stool visible in that quadrant, 1 is <half of the quadrant having stool, 2 is >half of the quadrant, and 3 is the entire quadrant filled with stool. Summing up all the quadrants, if the patient has more than 6 out of 12, the patient is deemed constipated.

Treatment of constipation uses a combination of non-pharmacological and pharmacological approaches [1]. If the patient is fit enough, they should be encouraged to drink fluids up to 1.5 L per day and mobilize more. The addition of prunes or prune juice, which have naturally occurring sorbitol to promote osmosis, is usually helpful and endorsed by patients. Fibre can be increased in patients who ingest inadequate amounts but not necessarily up to the usual recommended 30 g/day in this terminal population, as there is a risk of fecaloma or obstruction in those with abdominal malignancy and other comorbid factors [3]. In fact, some would recommend a low-residue diet to improve laxation [4]. The use of mineral oil per os and psyllium supplements are not recommended in this population. On the one hand, mineral oil will prevent lipid-soluble vitamins from being absorbed, and psyllium requires great quantities of fluids to be ingested to bulk up the stool enough to encourage a reflex peristaltic movement. As a result, psyllium is used for the treatment of diarrhea and fecal incontinence [5]. Privacy for defecation, proper positioning with knees higher up with feet on a step stool and maximizing on the natural morning peristaltic reflex can all encourage natural defecation.

The presence of stool in the rectosigmoid on the X-ray or on DRE will help dictate the next steps. The lower portion of the bowels needs to be evacuated first. Soft stool may require suppositories such as glycerine and/or bisacodyl. The bisacodyl suppository needs to touch the mucosa to provoke peristaltic movement. If suppositories are insufficient, a water enema can help by distending the bowel further and provoking a movement. To note that Fleet^R^ enemas are useful but, if used frequently, may cause some electrolyte imbalances. Hard stool may require manual disimpaction and/or an oil enema. Once the lower gut is empty of stool, oral laxatives can be started.

There are three major types of laxatives: stimulants, osmotics and mu-opioid receptor blockers. In a 2010 Canadian Consensus article [1], osmotics were the recommended laxatives. These include poly-ethylene glycol (PEG), lactulose, sorbitol and magnesium hydroxide. PEG is versatile as it can be mixed in any beverage or soup with minimal taste changes. Lactulose and sorbitol are very sweet and not tolerated by all; the author suggests dilution in water or other beverages for easier compliance. They can also cause abdominal gas and bloating. Magnesium hydroxide is not recommended in patients with renal failure as it can lead to hypermagnesemia. The stimulant laxatives include sennoside and bisacodyl, the latter being a stronger stimulant that occasionally causes abdominal cramping. Stimulants can be combined with osmotics if osmotic laxatives alone are insufficient to promote laxation. In patients taking regular dosing of opioids and in whom the constipation is not responsive to the above, a mu-receptor blocker can be added [2]. Two medications available in Canada fall in this category: methylnaltrexone, a subcutaneous medication, and naloxegol, which is in pill form. Neither molecule can penetrate the central nervous system and, therefore, do not affect analgesia [6]. Both can be expensive and so we suggest using them as adjunct medications on an as-needed basis. Some authors suggest switching the opioid route from per os to either the transdermal or parenteral routes, such as the subcutaneous route [2] to minimize their effect on the gut. Other medications that can be used as adjuncts are prucalopride (a 5HT4 serotonin receptors agonist which promotes peristaltism), linaclotide and lubiprostone (both increase intraluminal secretions) [7]. Again, these are expensive medications to be used as needed. Sodium docusate, a stool softener, is inadequate as a standalone medication and is no longer recommended.

Once the patient has their constipation issue resolved, it is important to keep them on a regular laxative regimen, which can be titrated up or downwards but should not be stopped entirely. It is important to remember to start a regular daily laxative prescription at the same time as daily opioids are initiated.

## 3. Nausea and Vomiting

Nausea and vomiting are also very common in patients with serious illnesses (20–60% of cancer patients [8]). They can be due to treatment (chemotherapy, radiotherapy), a complication of the illness (bowel obstruction, hypercalcemia, etc.), the illness itself and worsening of comorbidities such as renal failure. In a palliative population, the more frequent underlying pathophysiologies to provoke nausea are stimulation of the chemoreceptor trigger zone (CRTZ) and stimulation of the vagal nerve from the GI tract. Examples of triggers for the CRTZ are medications (opioids, chemotherapy), electrolyte abnormalities (hyponatremia, hypercalcemia), renal and hepatic failure and toxins from infections as well as those of progressing cancer. The nausea described by the patient will be an unrelenting nausea despite vomiting episodes. Nausea due to the GI tract can be secondary to severe thrush and medications (iron supplements, non-steroidal anti-inflammatories, steroids…) causing irritation of the mucosa, or, distension of the gut such as in gastroparesis (worsened by opioids), constipation, bowel obstruction and potentially hepatomegaly. This vagal-mediated nausea usually waxes and wanes, provoked by ingestion and relieved by vomiting. Less frequently, nausea can be due to increased intracranial pressure. In that case, the patient will often report accompanying headaches, with both symptoms being worse in the early morning when fluid redistribution will have caused the supine patient to experience worsened peritumoral cerebral edema. Stimulation of the vestibular system such as in neuroacoustic neuromas and cerebellar metastases will provoke a sensation of vertigo, which in turn will precipitate nausea. On rare occasions, opioids can cause this type of nausea as a side effect. A pathophysiology of exclusion is that of nausea provoked by high levels of emotions, such as fear, anxiety, and depression. An example can be that of anticipatory nausea before going to a chemotherapy session. All of these different mechanisms will report back to the Integrative Vomiting Centre in the brainstem; via the vagus nerve, the vomiting center will initiate vomiting. Multiple receptors at this level can be blocked to prevent vomiting.

As with all symptoms, a good assessment includes a comprehensive history searching for the provoking and alleviating factors of the nausea, associated symptoms, a physical exam focusing on the abdomen, neurological systems and to rule out thrush, and investigations (blood work and imaging) if warranted, to help understand the underlying pathophysiology and how to best address this troublesome symptom [9]. Depending on the goals of care of the patient, nausea and vomiting should be addressed by providing anti-emetics immediately, all the while trying to correct the underlying cause. Table 1 shows which receptors and medications are best for treating the various pathophysiological types of nausea. Some authors suggest using medications that will be effective in most cases of nausea, such as metoclopramide which crosses the blood–brain barrier thereby having an effect on the CRTZ, good in cases of increased intracranial pressure, in addition to the GI tract effect. It is worth noting that metoclopramide should be avoided in patients at risk of Parkinsonism and those with complete mechanical bowel obstruction [8]. Others are proponents of using medications that have activity on multiple receptors, enabling them to address most mechanisms of nausea [8,9,10]. Examples of such medications include olanzapine, mirtazapine and methotrimeprazine. Olanzapine with its long half-life can be given at bedtime and additionally addresses much of the limbic distress causing nausea [10]. Mirtazapine appears to be a promising drug for palliative patients, with anti-emetic, prokinetic, anti-depressant and anxiolytic activities. It possesses anti-dopaminergic, 5HT2, 5HT3, anti-histaminic and anti-muscarinic activities [9,10]. Patients with partially responding nausea should have a second anti-emetic added with an effect on a different receptor. Steroids such as dexamethasone and tetrahydrocannabinol (THC) could be third-line adjuncts, though the latter does not have much evidence to support its use [11]. Peripherally Restricted Opioid Receptor Antagonists (PAMORA) may have a role as anti-emetics for opioid-induced gastroparesis, whereas prucalopride (5-HT4 agonist), camicinal (motilin agonist), relamorelin (ghrelin agonist), gabapentin and ginger are all other avenues that require further study [8].

The Multinational Association of Supportive Care in Cancer 2021 consensus recommends metoclopramide and haloperidol as first-line treatments, methotrimeprazine and olanzapine as second-line and 5HT4 agonists and 5HT3 antagonists as third-line [12].

**Table 1:** Mechanism of nausea, the involved receptors and corresponding anti-emetics.

Mechanism	Receptors	Anti-Emetic	Comments
**Chemoreceptor trigger zone**	Dopamine 5HT3 Neurokinin 1	Haloperidol, Metoclopramide, Olanzapine, Methotrimeprazine Ondansetron Aprepitant	Risk of extrapyramidal side effects; risk of orthostatic hypotension with methotrimeprazine Constipation side effects with 5HT3 blockers
**Vagal** **—** **Gastrointestinal**	Dopamine 5HT3	Haloperidol, Metoclopramide, Olanzapine, Methotrimeprazine, Domperidone, Mirtazapine Ondansetron	Do not use metoclopramide in cases of complete bowel obstruction
**Increased intracranial pressure**	None specific	Haloperidol, Metoclopramide, Olanzapine, Methotrimeprazine	
**Vestibular**	Histamine Muscarinic	Mirtazapine, Methotrimeprazine, Dimenhydrinate Diphenhydramine Scopolamine	
**Limbic-cortex**	None specific	Cannabidiol, Olanzapine, Methotrimeprazine, Mirtazapine	
**Integrative Vomiting Centre**	5-HT2, 5-HT3 Muscarinic Histaminic CB1 Neurokinin 1	Olanzapine, Ondansetron Scopolamine Dimenhydrinate, Diphenhydramine, Methotrimeprazine, Mirtazapine Delta-9-tetrahydrocannabinol (THC) Aprepitant	

Adapted from Downing GM, Wainwright W, Victoria Hospice Society. Medical care of the dying, 4th edition. Victoria: Victoria Hospice Society, Learning Centre for Palliative Care; 2006 [13].

## 4. Ascites

Ascites can result from liver failure or peritoneal inflammation from intra-abdominal cancer propagation. When it is due to portal hypertension from cirrhosis or liver metastases, the renin–angiotensin–aldosterone system is being stimulated, whereas with most other malignant causes of ascites, obstructed lymphatics and altered vascular permeability are responsible for the accumulation of fluid [14]. The most common causes of ascites are ovarian cancer (25%), colorectal, pancreatic, uterine, gastric, peritoneal, lung, breast cancers and lymphoma [14]. There are a variety of symptoms that it can provoke: distension and abdominal pressure, constipation, orthopnea, dyspnea, and squashed stomach syndrome with early satiety, nausea and vomiting. Therefore, it is paramount to manage the volume of ascites to improve the overall QOL.

Diuretics, especially spironolactone in doses of 150 to 450 mg per day [15], can control small-volume ascites due to portal hypertension. When hyperkalemia prevents further increases in spironolactone, furosemide can be added. The maximal effect of diuretics may be seen after four weeks of treatment. Diuretics are less effective in the context of peritoneal carcinomatosis. The addition of midodrine (7.5 mg TID) to maintain blood pressure in cirrhotic patients on high diuretic dosing increases diuresis by improving renal perfusion [16]. Clonidine (0.1 mg BID) suppresses the renin–angiotensin–aldosterone system, thereby amplifying the effect of spironolactone in cirrhotic patients [16]. These are potential avenues to maximize the effects of diuretics in malignant ascites. Terlipressin, a vasopressin V1 receptor agonist, has vasoconstrictive effects on the splanchnic vessels resulting in improved systemic arterial blood pressure and renal perfusion, similar to clonidine and midodrine [16]. It is not yet approved for use in North America.

Octreotide was trialed [17], but studies have not been frankly positive in its efficacy and its cost is a deterrent. In a randomized double-blind study by Jatoi et al., patients who received long-acting octreotide experienced decreased bloating, abdominal discomfort and dyspnea, but still required ascites drainage [17]. The mechanism of action of octreotide is suspected to be through its inhibitory effect on vascular endothelial growth factor; this is abundantly produced by tumour cells by which they modify vascular permeability [15].

Abdominal paracentesis is an rapid and frequent solution. Repeat large-volume paracentesis carries risks of infection, bleeding, bowel perforation and hypotension, in addition to requiring frequent hospital visits [14,18]. Albumin replacement is usually offered to patients requiring large-volume (>4–5 L) paracentesis to prevent pre-renal and renal impairment from hypovolemia. Rapidly reaccumulating ascites should be managed with a permanent drain such as pigtail or PleurX. This will allow for the patient to have smaller volumes removed in a frequent fashion with fewer side-effects due to volume redistribution, better symptom control and the patient not needing to return to hospital [18]. Tunneled catheters (PleurX) lower infection risks [14,16].

Other methods of managing ascites are more invasive. Peritoneovenous shunts and transjugular intrahepatic portosystemic shunts carry a risk of complications such as infections, thrombophlebitis, disseminated intravascular coagulation and possible dissemination of metastases [14,15,17]. They are contra-indicated in the presence of liver failure, loculated ascites and positive cytology of ascitic fluid (high protein levels and red blood cells). Catumaxomab, a monoclonal antibody that binds to T cells, natural killer cells, macrophages and the epithelial adhesion molecule EpCAM, can be administered intraperitoneally to reduce ascites gradually over time. It targets EpCAM on tumour cells and decreases their number and effect on vascular permeability. Side effects include fever, nausea, ileus, infection, pleural effusions, and GI bleeds. A significant effect for minimizing ascites in cases of ovarian and other cancer types was shown [14,17]. Hyperthermic intraperitoneal chemotherapy is also a local way of managing ascites while mitigating systemic side effects. This intraperitoneal chemotherapy requires placement of inflow and outflow catheters which can be inserted perioperatively, laparoscopically or with ultrasound. Treatment is given every 4 to 6 weeks. There are risks of infection, perforation, fever, abdominal pain, formation of adhesions and bowel obstruction. However, some patients with diuretic-resistant malignant ascites may benefit from this form of management [14,15]. Select patients with longer prognoses could be considered for one of the above invasive procedures. Other novel therapies are described in the review on the management of malignant ascites by Smith and Jayson [15].

## 5. Gastro-Intestinal Bleeds

Gastro-intestinal bleeding can occur due to friable cancerous tissue being damaged in the gut, as complications of chemotherapy, radiotherapy, immunotherapy (bevacizumab), anticoagulation, secondary to thrombocytopenia, portal hypertension with varices, non-steroidal anti-inflammatories or steroid medications, and from other usual causes such as angiodysplasia of the intestine [19]. It is recommended to start prophylactic H2-blockers or proton pump inhibitors when starting a steroid regimen. Choosing a COXIB anti-inflammatory over other non-steroidal anti-inflammatories can decrease the chance of GI bleeding.

Initial management for patients not at end-of-life includes securing intravenous access, hemodynamic stabilization and investigation (complete blood count, coagulation profile, liver function tests, a CT scan or angiography). Consideration should be given to stopping blood thinners and other exacerbating factors. Transfusions and vitamin K can also be required.

A gastric or rectal bleed secondary to cancer can be stopped or minimized by local radiotherapy within 24 to 48 h [19]. Endoscopic procedures for upper GI and colorectal bleeds can stem the problem via cautery, injections of epinephrine, and laser or clip placement [19]. Portal hypertension and its complications will be minimized by the use of β-blocker anti-hypertensives. Octreotide can be initiated for its splanchnic vasoconstrictive activity. Tranexamic acid can be added to promote fibrin formation and clotting [19]. Tranexamic acid should be avoided in patients with a recent history of deep vein thrombosis, pulmonary embolus and unstable cardiovascular disease. Interestingly, hematologists have suggested the use of tranexamic acid concomitantly with low molecular weight heparin such as in the case of a recent thrombo-embolic disorder and a bleed [20].

Patients and family members must be advised of the risk that the bleed may turn into a catastrophic hemorrhage; dark towels at the bedside (to decrease the anxiety from seeing red blood on white sheets) as well as medication to decrease the patient’s anxiety should such a situation arise should be prescribed [19]. The author recommends a “distress protocol” of midazolam 5 mg given subcutaneously or intravenously every 10–20 min as needed to achieve sedation when the hemorrhage starts.

## 6. GI Obstructions

GI obstructions occur in up to 15% of cancer patients and are more commonly seen in colon cancers (10–29% of colon cancer patients) and ovarian cancers (20–50%) [2,21,22]. Other cancers that cause obstructions are stomach, pancreas, bladder, uterine, melanoma, lung and breast cancers [2,18,23,24]. They often occur in the context of very advanced illness, and as such require a palliative approach. The literature quotes prognoses varying from a few weeks to many months, the latter patients having received disease-modifying therapy and interventions [22,23,25]. Symptoms of GI obstruction may have been waxing and waning for weeks due to subocclusion prior to presenting with a complete obstruction [10,23,26,27]. Other than oesophageal obstructions, there are gastric outlet obstructions (GOO) and small and large bowel obstructions. Bowel obstructions are defined as: (1) clinical evidence of bowel obstruction, (2) bowel obstruction beyond the Ligament of Treitz, (3) intra-abdominal primary cancer with incurable disease, or (4) non-intra-abdominal primary cancer with intraperitoneal disease [21,25]. It is worth noting that small bowel obstructions (SBO) are more frequently encountered than large bowel obstructions (LBO) [24,26]: 61% vs. 33%.

Bowel obstructions can be due to mechanical or functional causes; it is important to differentiate them via a thorough investigation in order to manage the obstruction appropriately. History, physical exam and plain abdominal X-ray (two views) will confirm the presence of obstruction [18,28]. A CT scan of the abdomen will clarify whether this is a mechanical vs. a functional obstruction, the level of the obstruction, whether there are multiple levels of obstruction, differentiate a benign (adhesions, hernia, volvulus, strictures [25]) from a malignant cause and allow for an appreciation of the presence of ascites and larger peritoneal metastases [18]. MRI and PET scans do not provide more in terms of significant imaging [22]. All these findings have a role in deciding on the most appropriate treatment for the patient. Table 2 shows the symptoms and physical findings for esophageal, gastric-outlet, small bowel and large bowel obstructions. Treatment modalities will vary, from surgical options to the use of endoscopic interventions, and more conservative medical management. The decision to opt for one form of treatment over another will take into consideration the patient’s condition and estimated prognosis prior to the presentation of the obstruction as well as on presentation, the likely ability of the patient to withstand and benefit from the treatment and estimated functional recovery. This is especially important in the geriatric population, which tends to have multi-comorbities, worse stage and aggressive cancers [29]. The elderly population may tend to favour their autonomy and quality of life over increased survival time. Table 3 lists the patient factors which favour a surgical option in cases of mechanical obstruction, from those that are unfavourable. Regardless of the choice of treatment, symptom management should be initiated immediately. This allows for a wait-and-see approach, as there can be spontaneous resolution of the obstruction in 31–42% of cases [18,22,24,27] within approximately 7 days. On the other hand, evidence of bowel ischemia, perforation or closed-loop obstruction should warrant emergent surgery if the patient is fit and willing to undergo the procedure [23].

Nausea and vomiting are usually addressed by decompressing the gut with the use of a nasogastric (NG) tube [18]. However, the author has not had to resort to the use of NG tubes as the combination of anti-emetics and anti-secretory medications have controlled nausea and vomiting to an acceptable level for most patients [26]. If there is significant fluid loss due to vomiting, then intravenous or subcutaneous fluids should be started, especially in those patients with a longer prognosis where surgery is a consideration. However, this must be done cautiously to avoid worsening bowel wall edema and fluid build-up up-stream of the obstruction which would worsen symptoms [23]. All medications need to be reassessed, continuing the ones for symptom management via parenteral, transdermal or intra-rectal administration. Opioids will take care of the pain due to abdominal distension and cramping [18]. Anti-cholinergics will decrease colic further [18,26]. The patient is put on bowel rest, though they can have crushed ice as part of their mouth care.

Esophageal obstructions by malignancy will cause dysphagia. Palliation can be performed via stenting, dilation, or endoluminal brachytherapy (weekly radiotherapy administered locally by endoscopy), all of which carry significant risks of perforation, fistulas, as well as risks of re-obstructing [31]. A combination of stent and brachytherapy seems to yield better outcomes [32]. Stents should be avoided in patients who are still surgical or chemo-radiation therapy candidates as there could be a higher rate of future complications and mortality [32]. Other endoscopic temporary measures include cryotherapy, laser, alcohol or chemotherapy injections [31].

GOO can be treated surgically, with stenting, radiotherapy [33] or conservatively. As the majority of gastric cancers are advanced in the context of GOO, all treatments of GOO will have a palliative objective [34]. Many articles favour gastrojejunostomy (GJJ) for its longer patency, over metallic stenting which can re-obstruct within a matter of months. However, GJJ as a surgical intervention tends to cause significant numbers of complications. Laparoscopic and endoscopic ultrasound-guided GJJ might lower the risk of complications but may not be available in smaller hospitals [28,32,33,35,36]. In a retrospective study examining the impact of peritoneal carcinomatosis and ascites on survival and clinical success of de-obstructing with either GJJ or stent, survival and patency favoured the GJJ group, whether the patients had carcinomatosis and/or ascites or not. These findings did not always reach statistical significance [34]. From this we can deduce that GJJ should be reserved for patients younger than 50, with good functional status (ECOG 0–1), and a prognosis of greater than 2 months [32]. Stents are then recommended for those with a poor prognosis and recurrence of obstruction after having had surgery, but also as per the patient’s preference [34,36]. Stents offer a more rapid return of the per os route and discharge home but can re-obstruct in a median time of 67 days [33]. Others will receive medical management as described further.

In the fit patient, SBO and LBO can be treated surgically when bowel rest does not resolve the problem: resection, stoma formation and bypass. Resection offers a greater survival advantage (7 vs. 3 months) [24]; however, each type of surgery carries risks of affecting QOL and needs to be taken into account. Stoma formation is usually reserved for obstructions of the distal small bowel and large bowel, or if it is suspected that a colonic anastomosis would not heal well. Bypasses are used in patients with significant adhesions, where there was previous bowel irradiation, or where long lengths of bowel are involved [25]. The median survival after surgery for those patients with poor prognosis factors can be as short as 26 to 36 days but can be much longer (16 months) in select patients. Other reviews show 2.5 to 7.4 months of survival after surgery [22,30]. Re-obstruction rates after surgery can vary according to the presence of peritoneal carcinomatosis, up to 47%, and even 63% in ovarian cancers [18]. Surgery, especially open surgery, carries a high risk of complication which can vary from 5 to 87% depending on the study such as infections, thromboembolism, cardio-vascular, fistulas, dehiscence, pneumonia, urinary tract infections, anastomotic leaks and high-output ostomy [18]. Mortality is significant: 9 to 40% operatively, and 5 to 40% in the post-operative time [18].

Stents are also a possible option for right- and left-sided colon obstructions [18,25,32]. These yield high rates of immediate success and shorter hospital stays, but in the long term have risks of bowel perforation, migration and re-obstruction [25](40% of cases [27]). Stents also tend to be more effective in cases of intra-luminal rather than extra-luminal causes of obstruction, though they can still be attempted [25,32]. Stents are contra-indicated when there are multiple levels of obstruction, peritoneal carcinomatosis, perforation, and tumours within 5 cm of the rectum [18]. They can be considered as an option for patients with a prognosis of 1 to 2 months. Other endoscopic approaches such as laser ablation can provide temporary relief for rectal tumours [25].

Medical management of GI obstructions uses a combination of medications. There is much controversy over the use of steroids and octreotide [21,27,37] in the literature. Cochrane reviews usually conclude that not enough well-designed studies exist to come to a definitive conclusion or that the definition of success is not consistent across studies. Regardless, recommendations for the use of steroids persist [24,26]. Steroids decrease peritumoral edema in addition to gut wall inflammation. In doing so, the mass effect on the gut is decreased and may help restore the patency of the bowel. Additionally, by decreasing inflammation in the gut wall, there is decreased release of vasoactive intestinal peptide (VIP) and other inflammatory mediators. VIP, released by the mucosa under a hypertensive state, causes further electrolyte and water secretion into the gut upstream of the obstruction, which in turn causes more bowel distension and secondary nausea. Steroids have a role in breaking that cycle. Additionally, steroids have an analgesic and anti-emetic activity. The usual starting dose of dexamethasone is 8 to 16 mg per day in divided doses, at 8:00 am and noon, to avoid nighttime insomnia.

Octreotide is a somatostatin analogue. It has many interesting activities which are of benefit in obstructions. It blocks VIP release and decreases the production of gastric and pancreatic juices. Both actions make octreotide an anti-secretory medication that prevents further buildup of upstream volume [18,27]. It slows down peristalsis of the gut, thereby decreasing abdominal cramps [18] and minimizing the amount of bile released by the gallbladder, again, reducing the quantity of fluids upstream of the blockage. It is also vasoconstrictive in the gut, such that, along with a steroid, it can decrease peritumoral edema further in the hopes of reversing the obstruction. Other anti-secretagogues are H2-antihistamics, proton pump inhibitors, and anti-cholinergics such as hyoscine butylbromide for the reduction of gastric juices [27]. As the cost of octreotide is prohibitive, some have recommended first using hyoscine butylbromide with an H2-antihistaminic and reverting to the addition of octreotide in cases of uncontrolled nausea and vomiting [10,18,22]. Of note, it appears that H2-antihistaminics seem to be more effective at reducing gastric juices compared to proton pump inhibitors [22,23,24,37].

Control of nausea can usually be achieved with anti-emetics such as haloperidol, olanzapine, ondansetron [22,23], anti-histaminics and anti-cholinergics (hyoscine butylbromide) [27]. Should nausea and vomiting be non-remitting after three days’ worth of trying the above combination of medications as a continuous infusion at maximal doses, a venting gastrostomy tube placement should be considered [27]. NG tubes can cause mucosal injury, bleeding, reflux, esophagitis, aspiration pneumonia and sinusitis if they remain in place for a prolonged period of time [18,23,24,26]. A venting gastrostomy tube can be inserted by interventional radiology or gastroenterologists (by endoscopy) [25]. It allows the patient to eat and drink for pleasure, after which they empty their stomach contents. A relative contra-indication to the insertion of a gastrostomy tube is significant ascites but this can be mitigated by concomitant drainage [25,26,28], thereby minimizing peri-site leakage. Other relative contraindications include peritonitis, active gastric ulceration and coagulopathy [18].

Functional obstructions demarcate themselves from mechanical ones by their lack of colicky pain and bowel sounds. Cancerous tissue infiltrate bowel muscle, the mesentery, celiac or enteric plexus resulting in paralysis of the bowel [2,21,25,26]. Additionally, chemo- and radiation therapy, autonomic dysfunction, constipation, opioids, anti-cholinergics and electrolyte abnormalities can all lead to or worsen bowel dismotility [18,25]. One can use metoclopramide and erythromycin [38] to promote peristaltism in addition to steroids and osmotic laxatives [2]. Metoclopramide is also used in cases of subocclusion but never in complete mechanical obstruction.

In complete non-resolving GI obstruction, artificial hydration and nutrition can be contentious issues for patients, families and healthcare providers. Artificial hydration carries the risk of complications (increased ascites, pleural effusions, peripheral edema, bronchial secretions, nausea and vomiting) which can outweigh the limited benefits [26], as it does not improve survival nor symptoms of dehydration in the context of end-of-life [18,22,30]. Total parenteral nutrition (TPN) could be considered in a very select few who fulfill the following conditions: (i) slow-growing chemo-sensitive cancer who will receive more disease modifying treatment (ii) good performance status ECOG 0–1 (iii) no fluid retention (iv) no anemia (v) normal albumin levels [18]. TPN is more often reserved for patients who will undergo surgery followed by chemotherapy, to improve their nutritional status [18,22,24]. It is important to note that TPN improves QOL in only 25% of cases and has a high rate of complications in up to 54% of patients (catheter infections, deep vein thrombosis, hepatic dysfunction) [22]. Those patients whose obstruction reverses on medical therapy will gradually transition from a liquid to a solid low-residue diet, and will remain on long-term antisecretory medication (steroid or octreotide) to prevent a recurrence [18].

## 7. Conclusions

In conclusion, patients with serious illness are often prone to GI symptoms that significantly harm their QOL. Nurses, nurse practitioners, oncologists and family physicians who care for these patients should regularly assess this population for the presence of these symptoms. A cornerstone to their management is obtaining a good history, physical exam, investigations as warranted by the symptom and always making sure that the patient’s goals of care remain at the center of the management. Constipation can be managed with non-pharmacological approaches and with medications as tolerated by the patient. Nausea and vomiting can be addressed simultaneously with anti-emetics and by fixing the underlying cause where possible. Ascites can be acted upon early to prevent burdensome symptoms from developing through the use of diuretics but eventually may require more interventional techniques if and when the ascites become resistant. GI bleeds can in some cases come without warning and be catastrophic; other bleeds may come on gradually, allowing for medical treatment or interventions to control them. The patient and family need to be aware of all options, especially in the case of a GI obstruction, where the rate of complications and secondary burden can be high for some treatment options. Therefore, it is paramount that the patient understands and appreciates not just the underlying cause of their symptom(s), but also their prognosis. Regardless of the decision the team (doctors, nurse practitioner, surgeons, patient and family) may make, good palliation can provide comfort and QOL. It is now widely recognized that early palliative care involvement is helpful for the patients’ QOL by alleviating spiritual, social and psychological distress and for preventing physical symptoms or addressing them early on, as in the cases of constipation, ascites and nausea, as well as to explore goals of care. A multi-disciplinary approach will benefit the patient and their family in obtaining good symptom management and treatments that respect the patient’s values.

## Figures and Tables

**Table 2:** Level of GI obstruction with corresponding symptoms and clinical findings.

Level of Obstruction	Symptoms	Clinical Findings
Esophageal	Pain when swallowing, reflux of undigested food	
Gastric outlet obstruction	Nausea, projectile vomiting of food with clear gastric fluid	
Small bowel	Nausea, severe abdominal cramping mostly peri-umbilical or diffuse, vomiting with bile	Mild abdominal distension, high-pitch bowel sounds
Large bowel	Abdominal cramping more localized, constipation, no passing of gas, nausea late in presentation	Pronounced abdominal distension Possible mass on rectal exam

Table created by author.

**Table 3:** Predictors of surgical outcome.

Favourable	Unfavourable
Expected survival > 6 months	Poor prognosis < 3 months
ECOG 0–1	ECOG > 1
Benign cause of obstruction	
Localized disease	Stage 4 cancer
One level of obstruction	Multiple levels of obstruction
Age ≤ 45 years	Age > 65 years, frailty
Low histologic grade of cancer	
No or minimal nutritional deficiency	Malnutrition, cachexia, >9 kg weight loss, decreased albumin levels
No palpable abdominal mass	Palpable masses, abdominal carcinomatosis
No or little ascites < 3 L	Significant or recurrent ascites
No progression on prior chemotherapy	
No prior abdominal radiotherapy	Previous radiotherapy
	Elevated C-Reactive Protein, elevated white blood count
	Renal and/or hepatic failure
	Complete small bowel obstruction
	Non-gynecological cancer

From sources: [18,22,24,25,26,27,29,30].
